# The complete plastid genome of *Cheniella didyma* (H.Y.Chen) R.Clark & Mackinder (Leguminosae)

**DOI:** 10.1080/23802359.2021.1989333

**Published:** 2022-01-18

**Authors:** Qiubiao Zeng, Shiran Gu, Zhonghui Ma, Tieyao Tu

**Affiliations:** a National Demonstration Center for Experimental Plant Science Education, College of Agriculture, Guangxi University, Nanning, China; b Key Laboratory of Plant Resources Conservation and Sustainable Utilization, South China Botanical Garden, Chinese Academy of Sciences, Guangzhou, China; c University of Chinese Academy of Sciences, Beijing, China

**Keywords:** *Bauhinia*; *Cheniella*, chloroplast genome, Leguminosae, phylogeny

## Abstract

We report here for the first time the complete plastid genome of *Cheniella didyma* of the legume family. The plastid genome has a typical circular structure with a total length of 157,186 bp and contains two inverted repeat regions (IRs, 24,455 bp), a large single-copy region (LSC, 89,410 bp), and a small single-copy region (SSC, 18,866 bp). This is the first report of the complete plastid genome sequence of *Cheniella*, a genus recently segregated from *Bauhinia* s.l. The phylogenetic analysis based on 77 coding regions of the plastome of this species and those of the related species strongly suggested that *C. didyma* is sister to *Lysiphyllum* and is not directly related to *Bauhinia* s.s.

*Cheniella didyma* (H.Y. Chen) R.Clark & Mackinder 2017, a woody liana with pure white flowers blossom from July to October, is a beautiful species of the genus *Cheniella* in the legume family (Clark et al. 2017). It occurs exclusively in Guangdong Province and Guangxi Zhuang Autonomous Region of China. We herein assembled and annotated for the first time the complete plastome of *C. didyma* using a method of genomic sequencing to provide genetic and genomic information for further systematic and genetic researches.

Leaf tissues of *Cheniella didyma* were taken from Gaoshuikeng village, Enping, Guangdong Province, China (112.07E, 22.17N). The specimens (vouchers: TuTY4691_9 contact: qiub919@139.com) were deposited in the herbarium of South China Botanical Garden (IBSC), Guangzhou, China. We extracted the total genomic DNA by a modified CTAB method (Doyle and Doyle 1987). The isolated total genomic DNA was fragmented to make a library of 300-500 bp, and the paired-end sequences in length of *ca*. 150 bp were generated with Illumina (HiSeq X-Ten) at Beijing Genomics Institute (BGI) in Wuhan, China. The plastome was assembled by GetOrganelle pipeline (Bankevich et al. 2012; Langmead and Salzberg 2012; Wick et al. 2015; Jin et al. 2020), and Plastid Genome Annotator (PGA) (Qu et al. 2019) and Geneious (Kearse et al. 2012) were used to annotate and align the complete plastome. The annotated plastome has been deposited in GenBank (accession number: MZ230991).

To reconstruct the phylogenetic position of the species, we downloaded 13 plastid genome data of related species within Cercidoideae of the legume family from GenBank and used *Cercis* as outgroup to reconstruct the phylogenetic position of *Cheniella didyma* (Figure 1) (Sabir et al. 2014; Wang et al. 2017, 2018; Gu et al. 2019, 2020). We aligned the data matrix using MAFFT (Katoh and Standley 2013) as built in Geneious with default parameters. The phylogenetic relationship was estimated using the maximum likelihood method by RaxML-HPC2CIPRES Science Gateway (Miller et al. 2010) with models recommended by ModelFinder (Kalyaanamoorthy et al. 2017) based on a data matrix of concatenation of 77 coding regions (CDS). The branch supports were estimated using 1000 replicates of bootstrap. The complete plastid genome of *C. didyma* was 157,186 bp in length with a typical quadripartite structure: a large single copy (LSC) region of 89,410 bp and a small single copy (SSC) region of 18,866 bp, respectively. These two regions were separated by two inverted repeat regions (IRa and IRb), each of 24,455 bp in length. We recovered a total of 121 functional genes, including 80 protein-coding genes, 37 tRNA genes, and 4 rRNA genes. The overall GC content was 36.2%.

**Figure 1.**
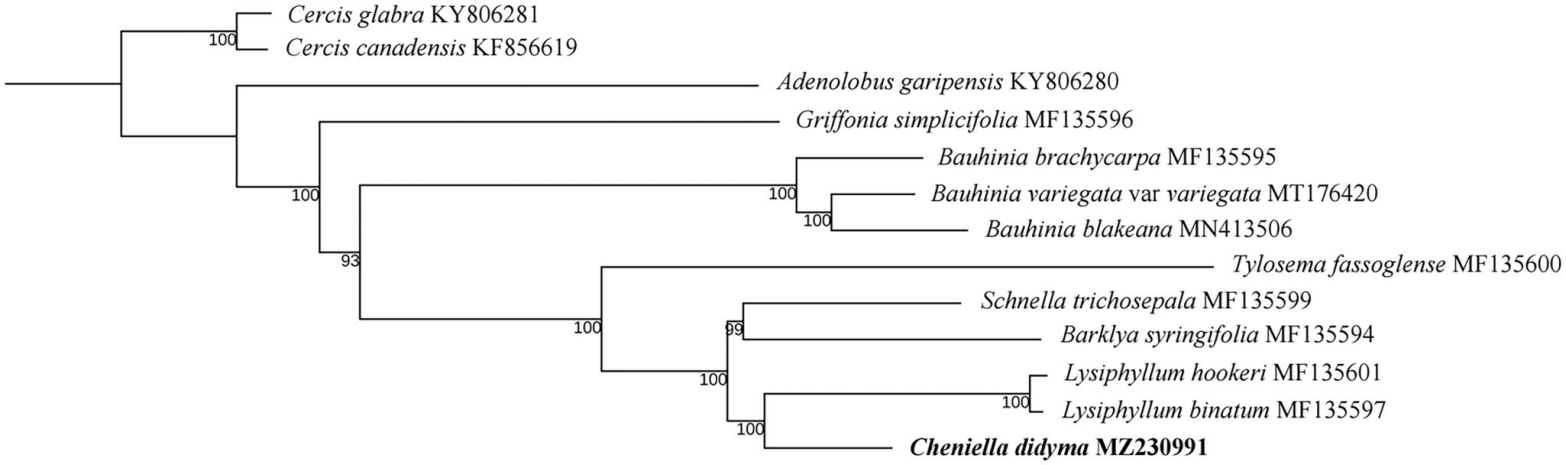
The maximum-likelihood (ML) phylogenetic tree based on 77 CDS of the plastid genomes. Numbers near the branches are bootstrap support values.

The phylogenetic analysis suggested that all the branches of the tree are strongly supported, suggesting the power of the plastid genome data in resolving the phylogenetic relationships within Cercidoideae. *Cheniella didyma* is recovered as a sister of *Lysiphyllum* and is not directly related to *Bauhinia* s.s, thus not conflicts with the treatment of *Cheniella* as a segregated genus by Clark et al. (2017). It may be expected that a comprehensive sampling covering more species of *Cheniella* and related taxa (especially *Phanera*) in future shall shed more light on the phylogenetic relationships of this plant group.

## Data Availability

The complete plastid genome of *Cheniella didyma* of this study is available in NCBI GenBank database (https://www.ncbi.nlm.nih.gov) with the accession number: MZ230991. The associated BioProject, SRA, and Bio-Sample numbers are PRJNA746089, SRR15130292, and SAMN20180496.
